# Construction and Analysis of the lncRNA-miRNA-mRNA Network Based on Competing Endogenous RNA in Atrial Fibrillation

**DOI:** 10.3389/fcvm.2022.791156

**Published:** 2022-01-24

**Authors:** Xiangyu Ke, Junguo Zhang, Xin Huang, Shuai Li, Meifang Leng, Zebing Ye, Guowei Li

**Affiliations:** ^1^Centre for Clinical Epidemiology and Methodology, Guangdong Second Provincial General Hospital, Guangzhou, China; ^2^Department of Epidemiology, School of Public Health, Sun Yat-sen University, Guangzhou, China; ^3^Department of Cardiology, Guangdong Second Provincial General Hospital, Guangzhou, China; ^4^Department of Health Research Methods, Evidence, and Impact (HEI), McMaster University, Hamilton, ON, Canada

**Keywords:** competing endogenous RNA, long non-coding RNAs, network, atrial fibrillation, bioinformatic analysis

## Abstract

**Background:**

Accumulated studies have revealed that long non-coding RNAs (lncRNAs) play critical roles in human diseases by acting as competing endogenous RNAs (ceRNAs). However, functional roles and regulatory mechanisms of lncRNA-mediated ceRNA in atrial fibrillation (AF) remain unknown. In the present study, we aimed to construct the lncRNA-miRNA-mRNA network based on ceRNA theory in AF by using bioinformatic analyses of public datasets.

**Methods:**

Microarray data sets of GSE115574 and GSE79768 from the Gene Expression Omnibus database were downloaded. Twenty-one AF right atrial appendage (RAA) samples and 22 sinus rhythm (SR) subjects RAA samples were selected for subsequent analyses. After merging all microarray data and adjusting for batch effect, differentially expressed genes were identified. Gene Ontology (GO) categories and Kyoto Encyclopedia of Genes and Genomes (KEGG) pathway enrichment analyses were carried out. A ceRNA network was constructed.

**Result:**

A total of 8 lncRNAs and 43 mRNAs were significantly differentially expressed with fold change >1.5 (*p* < 0.05) in RAA samples of AF patients when compared with SR. GO and KEGG pathway analysis showed that cardiac muscle contraction pathway were involved in AF development. The ceRNA was predicted by co-expressing LOC101928304/ LRRC2 from the constructional network analysis, which was competitively combined with miR-490-3p. The expression of LOC101928304 and LRRC were up-regulated in myocardial tissue of patients with AF, while miR-490-3p was down-regulated.

**Conclusion:**

We constructed the LOC101928304/miR-490-3p/LRRC2 network based on ceRNA theory in AF in the bioinformatic analyses of public datasets. The ceRNA network found from this study may help improve our understanding of lncRNA-mediated ceRNA regulatory mechanisms in the pathogenesis of AF.

## Introduction

Atrial fibrillation (AF) is the most common sustained arrhythmia characterized by irregular high-frequency excitation and contraction of the atria, and is a major contributor to stroke, heart failure and sudden death (1–3). Approximately 1–2% of the common population suffer from AF, with the prevalence up to over 10% for individuals aged ≥80 years (1, 2, 4, 5). However, there is a lack of effective therapy for AF in general, largely because the pathophysiologic mechanism underlying AF remains unclear. The underlying pathogenesis of AF is multiplex, including electrical remodeling, structural remodeling, Ca^2+^ handling abnormalities and autonomic nervous system changes (6).

Previous studies have identified that non-coding RNAs (ncRNAs) play a critical role in the pathogenesis of AF by regulating core proteins and pivotal pathways (7). Long non-coding RNAs (lncRNAs) are a class of endogenous ncRNAs whose length are >200 nucleotides with no protein-coding potential (8). A number of studies have demonstrated that lncRNAs are involved in multiple biological processes by regulating genes of transcriptional, posttranscriptional, and epigenetic levels (9). Dysregulations of lncRNA expressions and functions have been implicated in the development and progression of many diseases including cancer, neurodegeneration diseases and cardiovascular diseases (10–12).

Competing endogenous RNA (ceRNA) is a novel regulation mechanism in which lncRNA can competitively combine with miRNAs through microRNA response elements (MREs), thus inhibiting gene silencing by isolating miRNAs from messenger RNAs (mRNAs) (13–15). This regulation mechanism has been observed in some cardiovascular diseases. For example, lncRNA PEL was shown to function as a ceRNA to competitively band let-7d and contribute to cardiac fibrosis (16). In addition, lncRNA TNK2-AS1 could regulate ox-LDL-stimulated human aortic smooth muscle cell proliferation and migration by serving as a ceRNA for miR-150-50 to modulate VEGFA and FGF1 expression (17). However, the ceRNA mechanisms related to AF remained largely unclear and required further investigation.

Therefore, in the present study, we aimed to construct the lncRNA-miRNA-mRNA network based on ceRNA theory in AF by using bioinformatic analyses of public datasets. Findings from the ceRNA network may help improve our understanding of lncRNA-mediated ceRNA regulatory mechanisms in the pathogenesis of AF.

## Materials and Methods

### Microarray Datasets of mRNAs and LncRNAs

Two independent human AF gene expression profiles were downloaded from the Gene Expression Omnibus database (GEO, https://www.ncbi.nlm.nih.gov/geo) with accession number of GSE115574 and GSE79768, respectively (Table 1). All microarray data were based on GPL570 (Affymetrix Human Genome U133 Plus 2.0 Array). In each data set, only human right atrial appendage (RAA) samples from AF and sinus rhythm (SR) subjects were selected. Finally, a total of 21 AF and 22 SR samples were included for subsequent analyses.

**Table 1:** The characteristics of datasets in this study.

**GSE series**	**GSE115574**	**GSE79768**
Platform	GPL570	GPL570
Total	30	13
AF	14	7
SR	16	6
Country	Turkey	Taiwan
Contributors	Deniz GC et.al	Tsai F et.al

*GSE, gene expression omnibus; AF, atrial fibrillation; SR, sinus rhythm*.

### Data Pre-processing

Robust multi-array average (RMA) algorithm and log2-transformed were performed for background correction and normalization. The averages of the probe sets of values were calculated as the expression values for the same gene with multiple probe sets. Furthermore, human genome reference hg38 (GRCh38.p13) was utilized to annotate series matrix files to determine transcript biotype and to convert the probe IDs into gene symbols. After merging all microarray data, empirical Bayes frameworks was used to adjust for the batch effects.

### Differentially Expressed Genes Analysis

A differential expression analysis on merged GEO series based on paired-sample *t-*tests between AF and SR samples, were performed using “Limma” package of R software. The Benjamini and Hochberg (BH) method was introduced to adjust the raw *P*-values into a false discovery rate to avoid the multi-test problem. The adjust *p* < 0.05 and the gene expression fold change (FC) value ≥1.5 or ≤2/3 (|log2 FC| ≥ 0.58), were set as the thresholds for identifying DEGs including mRNA and LncRNA between the AF and SR sample. Moreover, up-regulated or down-regulated DEGs were visualized as heat map plots.

### Functional and Pathway Enrichment Analyses of DEGs Among ceRNA Network

To better understand the biological function and characteristics of DEGs among ceRNA network, Gene Ontology (GO, http://geneontology.org/) (18) and Kyoto Encyclopedia of Genes and Genomes (KEGG, https://www.kegg.jp/) (19) were performed using the “clusterProfiler” package of R software. GO terms and KEGG pathways that met the criterion of adjusted *p* < 0.05 and Q value <0.05, were considered as significantly enriched (18, 19).

### ceRNA Network Construction

To constructed LncRNA-miRNA-mRNA networks, first, the differentially expressed LncRNAs and mRNAs with |log2 FC| ≥0.58 were selected. The LncRNA-miRNA interactions were predicted by using Mircode (http://www.mircode.org), which provided human miRNA target predictions based on the comprehensive GENCODE gene annotation. Furthermore, predicted miRNAs were utilized to construct miRNA-mRNA interaction using the tools of TargetScan (http://www.targetscan.org/mamm_31/), Mirdb (http://mirdb.org/), MiRTarBase (http://miRTarBase.cuhk.edu.cn/), and MirDIP (http://ophid.utoronto.ca/mirDIP/). The mRNAs predicted by three or above datasets, were used to identify the potential miRNA targets. Since lncRNAs can function as endogenous miRNA sponges and regulate the translation of targeted mRNAs, the expression of lncRNAs and mRNAs should be positively correlated (14). Subsequently, the initial ceRNA network was constructed on the basis of the differentially expressed lncRNAs, predicted miRNAs and mRNAs using the Cytoscape 3.7.2 software (http://cytoscape.org/).

### Validation of the Expression of miRNAs Among ceRNA Network

In the ceRNA network, the expression of miRNAs should be negatively correlated with lncRNAs and mRNAs (14). The gene expression profile GSE28954 was downloaded from the GEO database and expression profiling arrays were generated using platform GPL10850 [Agilent-021827 Human miRNA Microarray (V3)]. Additionally, the GSE28954 dataset including 10 RAA sample (4 AF and 6 SR) was used to identify differential expression miRNAs. Any miRNAs were deemed as differentially expressed if adjusted *p*-values were < 0.05 and fold changes were ≥ 1.5 or ≤ 2/3. Subsequently, significantly down-regulated miRNAs, up-regulated mRNAs and LncRNAs were used to construct final ceRNA network. Moreover, Funrich software (http://www.funrich.org/) was used to functional enrichment for differential expression miRNAs.

## Results

### LncRNA and mRNA Expression Profile

**Figure 1:**
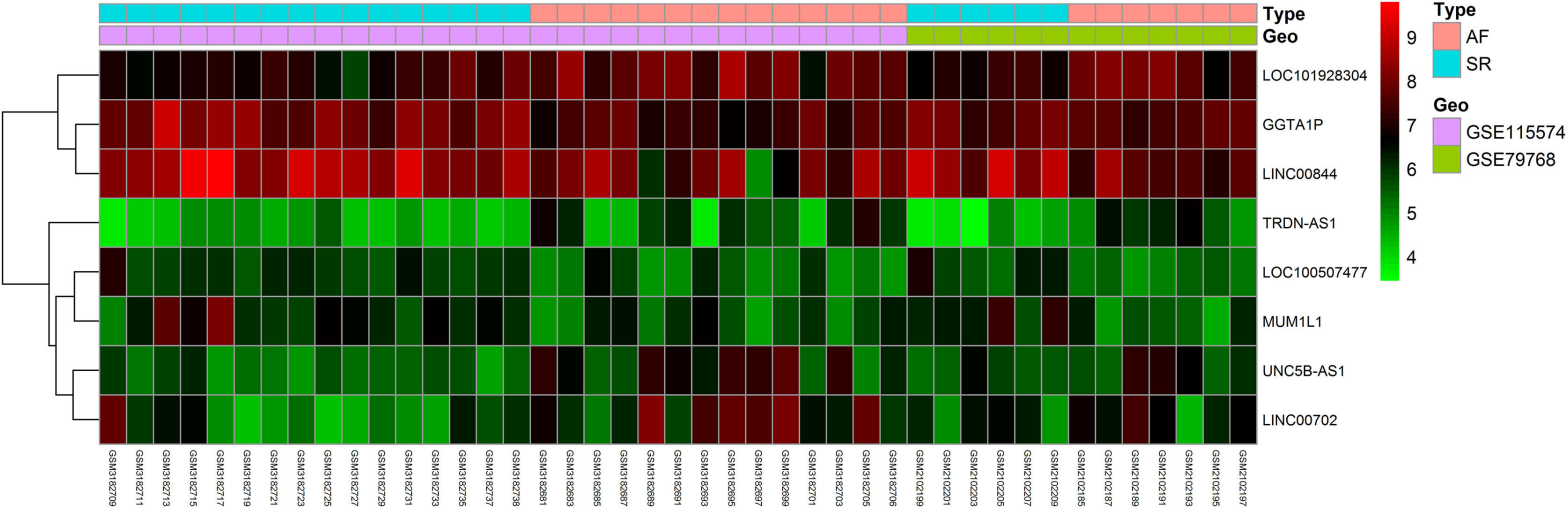
Heatmap of lncRNAs profile comparisons between atrial fibrillation and control. Each row represents a sample and each column represents a single gene. Pink color represents AF samples and blue color presents SR samples. The color scale shows the relative genes expression level in certain slide: green indicates low relative expression levels; red indicates high relative expression levels.

Gene expression levels of merged GEO series that have been adjusted for batch effects were standardized; and the results of pre- and post- standardized are showed in Supplementary Figure 1. A total of 8 lncRNAs and 43 mRNA were significantly differentially expressed with a fold change >1.5 (*p* < 0.05) in RAA samples of AF patients when compared with SR. The up-and down-regulated differently expressed lncRNAs and mRNAs are listed in Tables 2, 3. Among the 8 lncRNAs, 4 were up-regulated and the remaining were down-regulated. Among the 43 mRNAs, 14 and 39 were up-regulated and down-regulated, respectively. The heat maps of the 8 lncRNAs distinctly separated AF from controls were showed in Figure 1.

**Table 2:** The up-regulated and down-regulated differently expressed lncRNAs in merged data.

**LncRNA**	**FC**	**AveExpr**	**t**	***P* Value**	**Adjust *P* value**	**B**
**Up-regulated**						
*TRDN-AS1*	2.2933	5.0081	5.5285	<0.0001	0.0019	4.9934
*UNC5B-AS1*	1.9265	5.9359	4.8713	<0.0001	0.0066	3.0102
*LINC00702*	2.1401	6.1239	3.8955	0.0003	0.0323	0.1999
*LOC101928304*	1.6665	7.3767	4.8761	<0.0001	0.0066	3.0243
**Down-regulated**						
*LINC00844*	0.4813	8.1024	−4.7081	<0.0001	0.0089	2.5262
*MUM1L1*	0.5764	6.0297	−3.9736	0.0003	0.0286	0.4158
*LOC100507477*	0.6137	5.6490	−4.9845	<0.0001	0.0057	3.3483
*GGTA1P*	0.6632	7.7114	−4.8867	<0.0001	0.0065	3.0559

*FC, fold change; AveExpr, average expression, defined as average log2-expression level for that gene across all the arrays and channels in the experiment; B, The B-statistic is the log-odds given that the gene is differentially expressed*.

**Table 3:** The up-regulated and down-regulated differently expressed mRNA in merged data.

**mRNA**	**FC**	**AveExpr**	**t**	***P* Value**	**Adjust *P* value**	**B**
**Up-regulated**						
*DNAJA4*	1.5251	6.5447	7.2648	<0.0001	0.0001	10.2855
*DHRS9*	2.3195	8.4284	7.1253	<0.0001	0.0001	9.8654
*ANGPTL2*	1.5611	6.7732	6.2432	<0.0001	0.0005	7.1793
*FRMD3*	1.5410	7.8567	5.9835	<0.0001	0.0009	6.3838
*CHGB*	2.3222	7.9284	5.8236	<0.0001	0.0012	5.8944
*LBH*	1.7619	7.6184	5.6304	<0.0001	0.0017	5.3041
*RPL3L*	2.1094	7.2044	5.1515	<0.0001	0.0041	3.8500
*COLQ*	2.5338	6.1412	5.0006	<0.0001	0.0057	3.3964
*COL21A1*	1.5923	8.7335	4.7384	<0.0001	0.0089	2.6156
*LRRC2*	1.6346	6.6767	4.1833	0.0001	0.0212	1.0047
*PHLDA1*	1.7130	5.5968	4.1370	0.0002	0.0232	0.8736
*ATP1B4*	1.9225	4.0392	4.1332	0.0002	0.0232	0.8630
*RELN*	2.3546	7.1304	3.8306	0.0004	0.0358	0.0219
*HOGA1*	1.6400	5.9306	3.6332	0.0007	0.0469	−0.5107
**Down-regulated**						
*C1orf105*	0.4957	6.6784	−4.6479	<0.0001	0.0100	2.3488
*G0S2*	0.6187	9.3051	−4.6229	<0.0001	0.0105	2.2753
*SCARA5*	0.6410	7.2144	−4.5603	<0.0001	0.0120	2.0917
*TNNI1*	0.3292	6.7636	−4.4643	0.0001	0.0140	1.8121
*VIT*	0.5843	5.1497	−4.4554	0.0001	0.0141	1.7860
*BEX2*	0.5819	8.2081	−4.3737	0.0001	0.0160	1.5495
*PRIMA1*	0.6659	6.9603	−4.3647	0.0001	0.0160	1.5236
*CACNA2D2*	0.6092	7.1702	−4.2543	0.0001	0.0186	1.2069
*SFRP5*	0.5573	6.3132	−4.2466	0.0001	0.0186	1.1850
*SOSTDC1*	0.5526	5.3729	−4.2416	0.0001	0.0186	1.1706
*SYT13*	0.6506	4.9698	−4.2277	0.0001	0.0192	1.1311
*TMEM132C*	0.6557	6.1435	−4.1234	0.0002	0.0236	0.8353
*TMEM176A*	0.6291	8.0699	−4.0289	0.0002	0.0266	0.5700
*CNTN3*	0.5833	6.2783	−4.0121	0.0002	0.0275	0.5232
*IRX3*	0.6642	7.3042	−3.9392	0.0003	0.0306	0.3206
*MFAP4*	0.6284	7.9084	−3.8971	0.0003	0.0323	0.2042
*KCNN2*	0.6305	6.0473	−3.8486	0.0004	0.0349	0.0711
*ART3*	0.6384	8.6506	−3.8262	0.0004	0.0359	0.0098
*SUSD4*	0.6213	5.8862	−3.8150	0.0004	0.0362	−0.0208
*BCHE*	0.6113	6.4957	−3.7419	0.0005	0.0408	−0.2192
*CLSTN2*	0.6348	5.6674	−3.6521	0.0007	0.0459	−0.4605
*CPLX3*	0.5812	6.6010	−3.6176	0.0008	0.0485	−0.5522
*MGC24103*	0.6030	5.3526	−3.9094	0.0003	0.0316	0.2382
*REC114*	0.6487	4.9716	−5.8065	<0.0001	0.0012	5.8422
*TRDN*	0.6624	6.5037	−4.9370	<0.0001	0.0061	3.2060
*SLC7A11*	0.6358	4.7243	−4.8509	<0.0001	0.0069	2.9495
*BLM*	0.6175	6.1317	−4.8269	<0.0001	0.0073	2.8779

*FC, fold change; AveExpr, average expression, defined as average log2-expression level for that gene across all the arrays and channels in the experiment; B, The B-statistic is the log-odds given that the gene is differentially expressed*.

### Functional Enrichment Analyses: GO and KEGG Pathway Analyses

To further investigate the biological functions of the 43 differential expressed mRNA, functional enrichment analyses were performed (Table 4). GO enrichment analysis revealed that differential expressed mRNA were enriched in five biological processes (adjusted *p* < 0.05 and Q value < 0.05), but not in the AF associated process. Pathway analyses showed that differential expressed mRNA were enriched in 4 biological processes including bile secretion, cardiac muscle contraction, insulin secretion, protein digestion and absorption. The up-regulated ATP1B4 and down-regulated TRDN were enriched in cardiac muscle contraction (adjusted *p* = 0.0360 and Q = 0.0278). Cardiac muscle contraction was found to indicate AF development, which was line with a previous study (20).

**Table 4:** Significant enriched GO terms and pathways of differential expressed mRNA.

	**Term**	**Genes**	***P* value**	**Adjust *P* value**	**Q value**
**GO terms**					
GO:0001941	Postsynaptic membrane organization	RELN/SLC7A11/COLQ	<0.0001	0.0082	0.0057
GO:0043113	Receptor clustering	RELN/SLC7A11/COLQ	<0.0001	0.0107	0.0075
GO:0072578	Neurotransmitter-gated ion channel clustering	RELN/SLC7A11	0.0001	0.0157	0.0110
GO:0050807	Regulation of synapse organization	RELN/SLC7A11/COLQ/ CLSTN2	0.0001	0.0157	0.0110
GO:0050803	Regulation of synapse structure or activity	RELN/SLC7A11/COLQ/ CLSTN2	0.0001	0.0157	0.0110
**KEGG pathway**					
hsa04976	Bile secretion	ATP1B4/KCNN2	0.0028	0.0360	0.0278
hsa04260	Cardiac muscle contraction	TRDN/ATP1B4	0.0040	0.0360	0.0278
hsa04911	Insulin secretion	ATP1B4/KCNN2	0.0040	0.0360	0.0278
hsa04974	Protein digestion and absorption	COL21A1/ATP1B4	0.0048	0.0360	0.0278

### Construction of ceRNA Network

**Figure 2:**
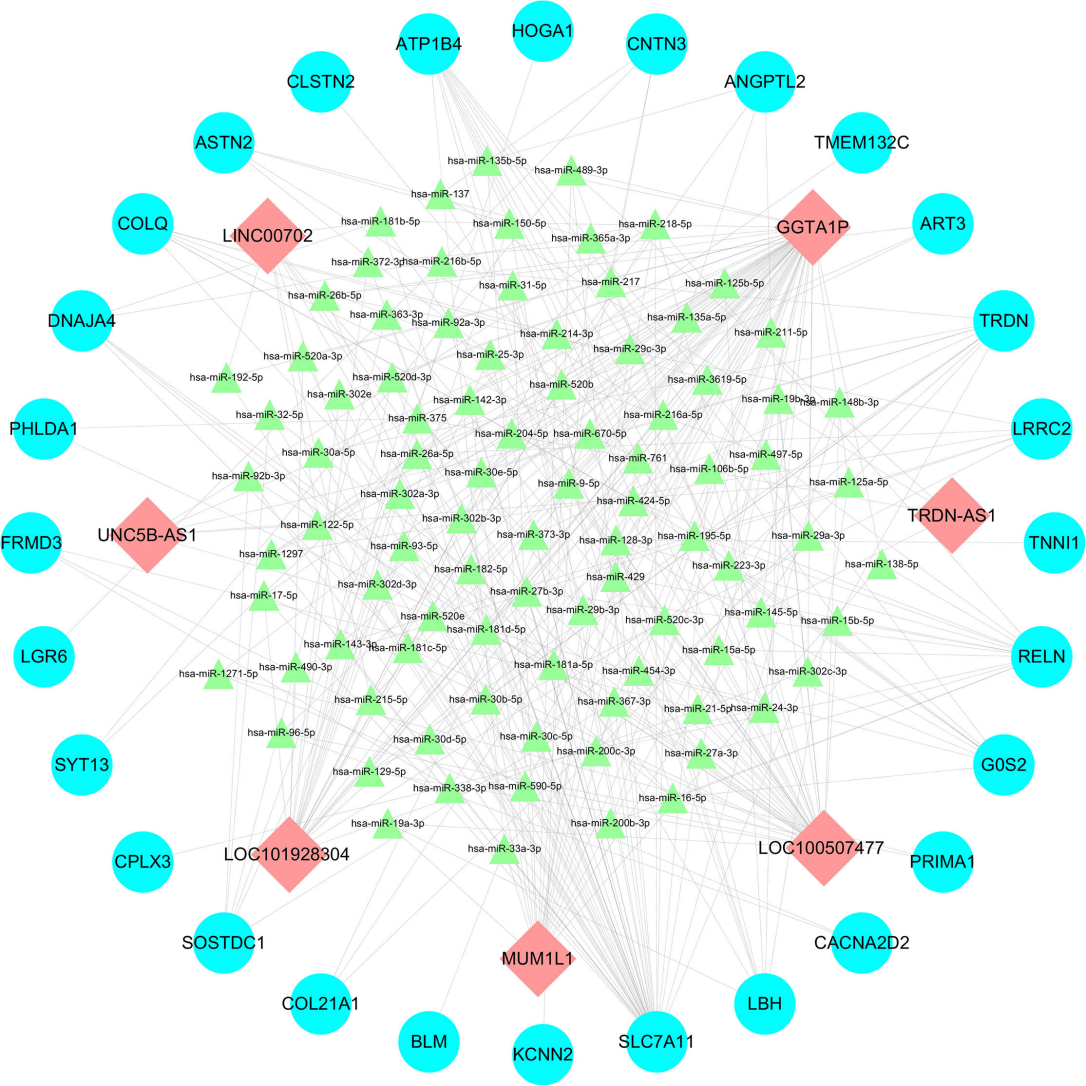
LncRNA-miRNA-mRNA networks between: Red, green, and blue represents lncRNAs, miRNAs and mRNAs, respectively.

Using the Mircode, we obtained 335 miRNAs. Furthermore, we obtained 77019 mRNAs based on the tools of TargetScan, Mirdb, MiRTarBase and MirDIP. By using the Cytoscape, we found that a total of 7 lncRNAs, 91 miRNAs and 28 mRNAs were involved in the lncRNA-miRNA-mRNA network (Figure 2). Based on the ceRNA theory (14), the screened lncRNAs and mRNAs were both up-regulated (Table 5).

**Table 5:** The ceRNA network of AF with up-regulated lncRNA and mRNA.

**lncRNA**	**miRNA**	**mRNA**	**lncFC**	**mFC**
UNC5B-AS1	hsa-miR-125a-5p	LBH	1.9265	1.7619
UNC5B-AS1	hsa-miR-125b-5p	LBH	1.9265	1.7619
LINC00702	hsa-miR-143-3p	LRRC2	2.1401	1.6346
LINC00702	hsa-miR-181a-5p	PHLDA1	2.1401	1.7130
LOC101928304	hsa-miR-204-5p	ANGPTL2	1.6665	1.5611
LOC101928304	hsa-miR-211-5p	ANGPTL2	1.6665	1.5611
LOC101928304	hsa-miR-214-3p	COLQ	1.6665	2.5338
LOC101928304	hsa-miR-218-5p	RELN	1.6665	2.3546
LINC00702	hsa-miR-27a-3p	RELN	2.1401	2.3546
LINC00702	hsa-miR-27b-3p	RELN	2.1401	2.3546
LOC101928304	hsa-miR-3619-5p	COLQ	1.6665	2.5338
LOC101928304	hsa-miR-375	PHLDA1	1.6665	1.7130
TRDN-AS1	hsa-miR-375	PHLDA1	2.2933	1.7130
LOC101928304	hsa-miR-490-3p	LRRC2	1.6665	1.6346
UNC5B-AS1	hsa-miR-670-5p	LBH	1.9265	1.7619
LOC101928304	hsa-miR-761	COLQ	1.6665	2.5338

*lncFC, lncRNA fold change; mFC, mRNA fold change*.

### Validation of the Expression of miRNAs Among ceRNA Network

To further validate the ceRNA network of AF, we analyzed miRNA microarrays GSE28954 from the GEO data and results showed that eight differentially expressed miRNAs were identified (Table 6). After combining with the findings from Tables 5, 6, we found that LOC101928304/miR-490-3p/LRRC2 was consistent with the ceRNA mechanism. The expression of LOC101928304 and LRRC2 were up-regulated in myocardial tissue of patients with AF, while miR-490-3p was down-regulated. LOC101928304 was positively correlated with LRRC2 (Supplementary Figure 2).

**Table 6:** The differential expression of miRNA in AF patients compared with controls.

**miRNA**	**FC**	**logFC**	**AveExpr**	**t**	***P*-Value**	**adj.P.Val**	**B**
hsa-miR-146b-5p	5.2289	2.3865	0.1283	10.4588	0.0000	0.0005	5.8165
hsa-miR-21-5p	3.3088	1.7263	−0.3083	5.5215	0.0002	0.0608	0.9038
hsa-miR-331-3p	0.6302	−0.6661	−0.0134	−5.0775	0.0005	0.0768	0.2909
hsa-miR-490-3p	0.3849	−1.3774	−0.1368	−4.6268	0.0009	0.0768	−0.3638
hsa-miR-139-5p	0.5946	−0.7500	−0.0444	−4.6021	0.0009	0.0768	−0.4007
hsa-miR-125b-2-3p	0.6196	−0.6906	0.0241	−4.5304	0.0011	0.0768	−0.5080
hsa-miR-143-5p	0.4905	−1.0278	−0.2194	−4.5209	0.0011	0.0768	−0.5223
hsa-miR-128-3p	0.6406	−0.6424	−0.0031	−4.2403	0.0017	0.0930	−0.9502

*FC, fold change; Log_2_FC, log_2_Fold change; AveExpr, average expression, defined as average log2-expression level for that gene across all the arrays and channels in the experiment; B, The B-statistic is the log-odds given that the gene is differentially expressed*.

## Discussion

In this bioinformatics study, we integrated gene expression profiles of 46 AF samples and 31 SR samples from 2 GEO datasets. We identified 8 lncRNAs and 43 mRNA were significantly differentially expressed with fold change >1.5 (*p* < 0.05). Furthermore, the LOC101928304/miR-490-3p/LRRC2 network based on ceRNA theory was constructed in AF. The expression of LOC101928304 and LRRC2 were up-regulated in myocardial tissue of patients with AF, while miR-490-3p was down-regulated.

Recently, the pathological functions of lncRNAs and miRNAs in cardiovascular disease have been recognized, making them potential candidates as therapeutic targets and biomarkers. However, AF-related lncRNAs and miRNAs still required further research. Recent studies have investigated the expression profiles of lncRNA in AF patients (20–25). For instance, Xu et al. detected the expression levels of lncRNAs in AF in elderly patients, which showed that lncRNAs were closely involved in the pathogenesis of AF (26). Moreover, an increasing number of reports indicate that lncRNAs can regulate protein-coding genes in mammals through the ceRNA network (27), where lncRNAs function as miRNA sponges to upregulate the expression of their targets (28). For instance, Liu et al. identified LINC00964 as a key lncRNA in AF susceptibility- and persistence-associated ceRNA networks (29). By integrating three microarray datasets and construction of a ceRNA network, the study by Wu et al. discovered that HCG11, KRBOX1-AS1, ACBD5 and RAD52 may compete with WEE1 for has-miR-17-5p to affect the development of AF (30). However, given the sparse existing evidence in the literature, the ceRNA mechanisms related to AF remained largely obscure and required further investigation.

Previous studies have found an association between LOC101928304 and cardiovascular diseases. Qiu et al. found that LOC101928304 were related with the functions of blood circulation and heart contraction using high-throughput sequencing of lncRNAs in myocardial tissues collected from patients with dilated cardiomyopathy (DCM) and normal heart donors (31). They also confirmed that LOC101928304 were downregulated in DCM tissues by using real-time PCR (31). Likewise, one study reported that miR-490-3p played a key role in different acute and chronic cardiovascular disease processes (32). Downregulation of miR-490-3p expression in atherosclerosis patients, and miR-490-3p which enhanced cell proliferation and migration of vascular smooth muscle cells and human umbilical vein endothelial cells in atherosclerosis (33, 34). The inhibition of miR-490-3p could promote autophagy to reduce myocardial ischemia reperfusion (IR) injury by upregulating ATG4B (35). Qiang et al. investigated that miR-490-3p was downregulated in patients with chronic heart failure (HF), which might provide novel targets for prevention and treatment of chronic HF (36). Cao et al. revealed that miR-490-3p was down-regulated in AF, which had a potential diagnostic value to distinguish patients with AF from healthy controls (37). First, we constructed the ceRNA network based on lncRNA and mRNA, and predicted the corresponding miRNA. In subsequent verification, we carried out an exploratory analysis because the sample size was limited. Under these circumstances, even if its adjusted *P* > 0.05, we still have reason to believe that miR-490-3p has potential to be part of the ceRNA network that could be combined with published literature, which should be further verified in subsequent experimental studies. Taken together, our findings were consistent with results from previous studies in that miR-490-3p may participate in the ceRNA network of LOC101928304.

LRRC2 located in chromosome three common eliminated region one, is a member of the leucine-rich repeat-containing family of proteins that have been implicated in various biological pathways. Recently, LRRC2 has been reported to correlate with transcripts associated with cardiac remodeling, thereby playing critical roles in the processes of cardiomyocyte hypertrophy and mitochondrial abundance (38). RNAi-mediated LRRC2 knockdown in a rat-derived cardiomyocyte cell line resulted in enhanced expression of canonical hypertrophic biomarkers as well as increased mitochondrial mass in the context of increased PGC-1α expression (38). LRRC2 partially localized to the mitochondrion and regulated by the mitochondrial master regulator PGC-1α (38). Most notably, mitochondrial dysfunction and cardiomyocyte hypertrophy increase the heterogeneity of atrial electrical conduction, leading to change in atrial structure and potentially facilitating the development of AF (39). The expression of PGC-1α was significantly decreased in AF model of rabbits with rapid pacing, indicating that mitochondrial biosynthesis was impaired in AF (40). LRRC2 may be a mediator of mitochondrial and cardiac function, which involve the PGC-1α-dependent mitochondrial abundance regulation mechanism, thereby ultimately facilitating the development of AF. Nevertheless, a more thorough understanding of LOC101928304/miR-490-3p/LRRC2 pathway in AF is necessary.

Discovering ncRNA-disease associations may help generate diagnostic and therapeutic tools for diseases. However, since uncovering associations via experimental studies are resource and time consuming, bioinformatic analyses for the identification of ncRNAs associated with AF are a helpful approach for preliminary exploration. In our study, we constructed a novel lncRNA-miRNA-mRNA network (LOC101928304/miR-490-3p/LRRC2) based on the ceRNA theory, which may therefore provide insights into the ceRNA regulatory mechanisms in the pathogenesis of AF. However, our study has some limitations. First, it is difficult to integrate some important factors for analyses, including regions, races and age. Given that the development of AF was caused by numerous environmental and genetic factors, our findings may be influenced by some immeasurable risk factors to an unknown extent. Additionally, specimens were obtained only from within the RAA, whereas AF is predominantly a left atrial disease. KEGG pathway analyses showed that the differently expression lncRNAs were mainly associated with cardiac muscle contraction. The up-regulated ATP1B4 and down-regulated TRDN were enriched in cardiac muscle contraction. Therefore, LOC101928304/miR-490-3p/LRRC2 may be involved in AF through other pathways. Our study may provide some new insights into the AF mechanism based on the bioinformatics analyses; however, the significantly differentially expressed lncRNAs and mRNAs would require experimental investigation of RT-PCR in clinical samples for further validation. In addition, the significantly differentially expressed lncRNAs require experimental investigations using the left atria appendage/pulmonary vein region samples for further validation. Likewise, future experimental research is needed to validate the ceRNA network of LOC101928304/miR-490-3p/LRRC2 in the pathogenesis of AF.

## Conclusion

The LOC101928304/miR-490-3p/LRRC2 network based on ceRNA theory was constructed in AF in this bioinformatic analysis study. The novel ceRNA network found from this study may help improve our understanding of lncRNA-mediated ceRNA regulatory mechanisms in the pathogenesis of AF.

## Data Availability Statement

The datasets presented in this study can be found in online repositories. The names of the repository/repositories and accession number(s) can be found in the article/Supplementary Material.

## Author Contributions

XK, JZ, ZY, and GL were responsible for study conception, design of the study, data acquisition, and analysis and interpretation of results. ML was responsible for data acquisition. ML, XH, and SL took part in the discussion of the paper. XK wrote the manuscript that was reviewed and revised by GL, ZY, ML, XH, and SL. All authors gave approval of the version to be submitted.

## Funding

This work was supported by the Medical Scientific Research Foundation of Guangdong Province of China (Grant sponsor: GL; Grant no. A2020453), the Science Foundation of Guangdong Second Provincial General Hospital (Grant sponsor: GL; Grant no.YY2018-002), and Doctoral workstation foundation of Guangdong Second Provincial general Hospital (Grant sponsor: XK; Grant no. 2021BSGZ008).

## Conflict of Interest

The authors declare that the research was conducted in the absence of any commercial or financial relationships that could be construed as a potential conflict of interest.

## Publisher's Note

All claims expressed in this article are solely those of the authors and do not necessarily represent those of their affiliated organizations, or those of the publisher, the editors and the reviewers. Any product that may be evaluated in this article, or claim that may be made by its manufacturer, is not guaranteed or endorsed by the publisher.

## References

[1] Ahmad Y Lip GY Lane DA. Recent developments in understanding epidemiology and risk determinants of atrial fibrillation as a cause of stroke. Can J Cardiol. (2013) 29:S4–13. 10.1016/j.cjca.2013.03.009 23790597

[2] Andrade J Khairy P Dobrev D Nattel S. The clinical profile and pathophysiology of atrial fibrillation: relationships among clinical features, epidemiology, and mechanisms. Circ Res. (2014) 114:1453–68. 10.1161/CIRCRESAHA.114.303211 24763464

[3] Luo X Yang B Nattel S. MicroRNAs and atrial fibrillation: mechanisms and translational potential. Nat Rev Cardiol. (2015) 12:80–90. 10.1038/nrcardio.2014.178 25421165

[4] Naccarelli GV Varker H Lin J Schulman KL. Increasing prevalence of atrial fibrillation and flutter in the United States. Am J Cardio. (2009) 104:1534–9. 10.1016/j.amjcard.2009.07.022 19932788

[5] Murphy NF Simpson CR Jhund PS Stewart S Kirkpatrick M Chalmers J. A national survey of the prevalence, incidence, primary care burden and treatment of atrial fibrillation in Scotland. Heart. (2007) 93:606–12. 10.1136/hrt.2006.107573 17277353 PMC1955558

[6] Nattel S Harada M. Atrial remodeling and atrial fibrillation: recent advances and translational perspectives. J Am Coll Cardiol. (2014) 63:2335–45. 10.1016/j.jacc.2014.02.555 24613319

[7] Qian C Li H Chang D Wei B Wang Y. Identification of functional lncRNAs in atrial fibrillation by integrative analysis of the lncRNA-mRNA network based on competing endogenous RNAs hypothesis. J Cell Physiol. (2019) 234:11620–30. 10.1002/jcp.27819 30478836

[8] Liu X She Y Wu H Zhong D Zhang J. Long non-coding RNA Gas5 regulates proliferation and apoptosis in HCS-2/8 cells and growth plate chondrocytes by controlling FGF1 expression via miR-21 regulation. J Biomed Sci. (2018) 25:18. 10.1186/s12929-018-0424-6 29490650 PMC5830091

[9] Kornienko AE Guenzl PM Barlow DP Pauler FM. Gene regulation by the act of long non-coding RNA transcription. BMC Biol. (2013) 11:59. 10.1186/1741-7007-11-59 23721193 PMC3668284

[10] Camacho CV Choudhari R Gadad SS. Long noncoding RNAs and cancer, an overview. Steroids. (2018) 133:93–5. 10.1016/j.steroids.2017.12.012 29317255

[11] Yu WD Wang H He QF Xu Y Wang XC. Long noncoding RNAs in cancer-immunity cycle. J Cell Physiol. (2018) 233:6518–23. 10.1002/jcp.26568 29574911 PMC13482439

[12] Shi Q Yang X. Circulating microrna and long noncoding rna as biomarkers of cardiovascular diseases. J Cell Physiol. (2016) 231:751–5. 10.1002/jcp.25174 26308238

[13] Karreth FA Pandolfi PP. ceRNA cross-talk in cancer: when ce-bling rivalries go awry. Cancer Discov. (2013) 3:1113–21. 10.1158/2159-8290.CD-13-0202 24072616 PMC3801300

[14] Tay Y Rinn J Pandolfi PP. The multilayered complexity of ceRNA crosstalk and competition. Nature. (2014) 505:344–52. 10.1038/nature12986 24429633 PMC4113481

[15] An Y Furber KL Ji S. Pseudogenes regulate parental gene expression via ceRNA network. J Cell Mol Med. (2017) 21:185–92. 10.1111/jcmm.12952 27561207 PMC5192809

[16] Liang H Pan Z Zhao X Liu L Sun J Su X. LncRNA PFL contributes to cardiac fibrosis by acting as a competing endogenous RNA of let-7d. Theranostics. (2018) 8:1180–94. 10.7150/thno.20846 29464008 PMC5817119

[17] Cai T Cui X Zhang K Zhang A Liu B Mu JJ. LncRNA TNK2-AS1 regulated ox-LDL-stimulated HASMC proliferation and migration via modulating VEGFA and FGF1 expression by sponging miR-150-5p. J Cell Mol Med. (2019) 23:7289–98. 10.1111/jcmm.14575 31468685 PMC6815783

[18] Ashburner M Ball CA Blake JA Botstein D Butler H Cherry JM. Gene ontology: tool for the unification of biology. The gene ontology consortium. Nat Genet. (2000) 25:25–9. 10.1038/75556 10802651 PMC3037419

[19] Kanehisa M Sato Y Furumichi M Morishima K Tanabe M. New approach for understanding genome variations in KEGG. Nucleic Acids Res. (2019) 47:D590–5. 10.1093/nar/gky962 30321428 PMC6324070

[20] Wu N Li J Chen X Xiang Y Wu L Li C. Identification of long non-coding RNA and circular RNA expression profiles in atrial fibrillation. Heart Lung Circ. (2020) 29:e157–67. 10.1016/j.hlc.2019.10.018 31843366

[21] Ruan Z Sun X Sheng H Zhu L. Long non-coding RNA expression profile in atrial fibrillation. Int J Clin Exp Pathol. (2015) 8:8402–10. 26339410 PMC4555738

[22] Su Y Li L Zhao S Yue Y Yang S. The long noncoding RNA expression profiles of paroxysmal atrial fibrillation identified by microarray analysis. Gene. (2018) 642:125–34. 10.1016/j.gene.2017.11.025 29129807

[23] Yu XJ Zou LH Jin JH Xiao F Li L Liu N. Long noncoding RNAs and novel inflammatory genes determined by RNA sequencing in human lymphocytes are up-regulated in permanent atrial fibrillation. Am J Transl Res. (2017) 9:2314–26. 28559982 PMC5446514

[24] Mei B Liu H Yang S Liang MY Yue Y Huang SQ. Long non-coding RNA expression profile in permanent atrial fibrillation patients with rheumatic heart disease. Eur Rev Med Pharmacol Sci. (2018) 22:6940–7. 10.26355/eurrev_201810_16165 30402860

[25] Ruan ZB Wang F Gongben BD Chen GC Zhu L. Identification of circulating lncRNA expression profiles in patients with atrial fibrillation. Dis Markers. (2020) 2020:8872142. 10.1155/2020/8872142 33299500 PMC7704132

[26] Xu Y Huang R Gu J Jiang W. Identification of long non-coding RNAs as novel biomarker and potential therapeutic target for atrial fibrillation in old adults. Oncotarget. (2016) 7:10803–11. 10.18632/oncotarget.7514 26908457 PMC4905440

[27] Chu Q Xu T Zheng W Chang R Zhang L. Long noncoding RNA MARL regulates antiviral responses through suppression miR-122-dependent MAVS downregulation in lower vertebrates. PLoS Pathog. (2020) 16:e1008670. 10.1371/journal.ppat.1008670 32678830 PMC7390449

[28] Du Z Sun T Hacisuleyman E Fei T Wang X Brown M. Integrative analyses reveal a long noncoding RNA-mediated sponge regulatory network in prostate cancer. Nat Commun. (2016) 7:10982. 10.1038/ncomms10982 26975529 PMC4796315

[29] Liu Y Liu N Bai F Liu Q. Identifying ceRNA networks associated with the susceptibility and persistence of atrial fibrillation through weighted gene co-expression network analysis. Front Genet. (2021) 12:653474. 10.3389/fgene.2021.653474 34249084 PMC8261127

[30] Wu J Deng H Chen Q Wu Q Li X Jiang S. Comprehensive analysis of differential immunocyte infiltration and potential ceRNA networks involved in the development of atrial fibrillation. Biomed Res Int. (2020) 2020:8021208. 10.1155/2020/8021208 33015181 PMC7525288

[31] Qiu Z Ye B Yin L Chen W Xu Y Chen X. Downregulation of AC061961.2, LING01-AS1, and RP11-13E1.5 is associated with dilated cardiomyopathy progression. J Cell Physiol. (2019) 234:4460–71. 10.1002/jcp.27247 30203513

[32] Fan ZX Yang J. The role of microRNAs in regulating myocardial ischemia reperfusion injury. Saudi Med J. (2015) 36:787–93. 10.15537/smj.2015.7.11089 26108581 PMC4503896

[33] Liu Y Chen Y Tan L Zhao H Xiao N. Linc00299/miR-490-3p/AURKA axis regulates cell growth and migration in atherosclerosis. Heart Vessels. (2019) 34:1370–80. 10.1007/s00380-019-01356-7 30734057

[34] Guo X Liu Y Zheng X Han Y Cheng J. HOTTIP knockdown inhibits cell proliferation and migration via regulating miR-490-3p/HMGB1 axis and PI3K-AKT signaling pathway in ox-LDL-induced VSMCs. Life Sci. (2020) 248:117445. 10.1016/j.lfs.2020.117445 32081664

[35] Wu Y Mao Q Liang X. Targeting the MicroRNA-490-3p-ATG4B-Autophagy axis relieves myocardial injury in ischemia reperfusion. J Cardiovasc Transl Res. (2021) 14:173–83. 10.1007/s12265-020-09972-9 32474761

[36] Qiang L Hong L Ningfu W Huaihong C Jing W. Expression of miR-126 and miR-508-5p in endothelial progenitor cells is associated with the prognosis of chronic heart failure patients. Int J Cardiol. (2013) 168:2082–8. 10.1016/j.ijcard.2013.01.160 23465244

[37] Cao Y Cui L. Identifying the key microRNAs implicated in atrial fibrillation. Anatol J Cardiol. (2021) 25:429–36. 10.14744/AnatolJCardiol.2020.41625 34100730 PMC8210928

[38] McDermott-Roe C Leleu M Rowe GC Palygin O Bukowy JD Kuo J. Transcriptome-wide co-expression analysis identifies LRRC2 as a novel mediator of mitochondrial and cardiac function. PloS oNE. (2017) 12:e0170458. 10.1371/journal.pone.0170458 28158196 PMC5291451

[39] Neuberger HR Schotten U Verheule S Eijsbouts S Blaauw Y van Hunnik A. Development of a substrate of atrial fibrillation during chronic atrioventricular block in the goat. Circulation. (2005) 111:30–7. 10.1161/01.CIR.0000151517.43137.97 15630037

[40] Dong J Zhao J Zhang M Liu G Wang X Liu Y. Beta3-adrenoceptor impairs mitochondrial biogenesis and energy metabolism during rapid atrial pacing-induced atrial fibrillation. J Cardiovasc Pharmacol Ther. (2016) 21:114–26. 10.1177/1074248415590440 26130614 PMC5810932

