# Supplementary material for: Construction and Analysis of the lncRNA-miRNA-mRNA Network Based on Competing Endogenous RNA in Atrial Fibrillation
Source: Front Cardiovasc Med. 2022 Jan 24;9:791156. doi: 10.3389/fcvm.2022.791156 (PMC8818759; doi:10.3389/fcvm.2022.791156)
Supplement: Supplementary file 1 [file Data_Sheet_1.pdf]

Supplementary Figures

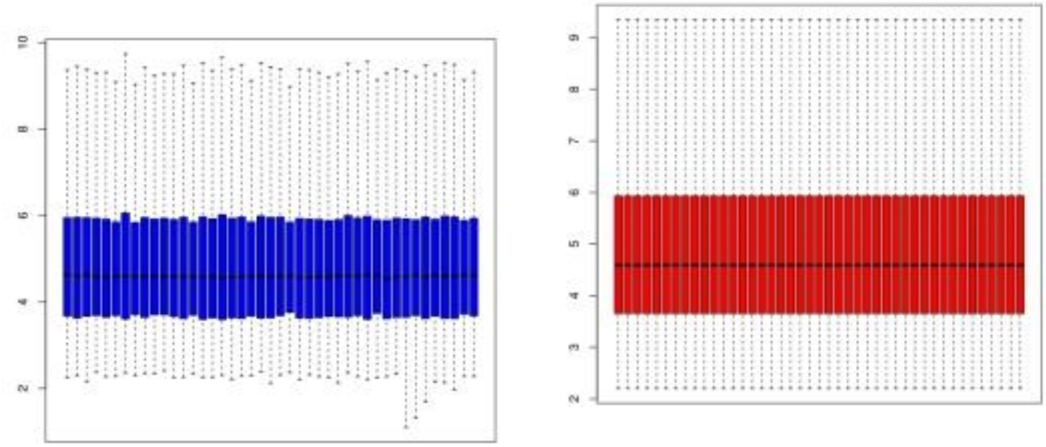

(a) (b)

Supplementary Figure 1 (a) Data standardization. Pre-standardization gene expression levels of each data set are presented as blue boxplots; (b) Data standardization. Post-standardization gene expression levels of each data set are presented as red boxplots.

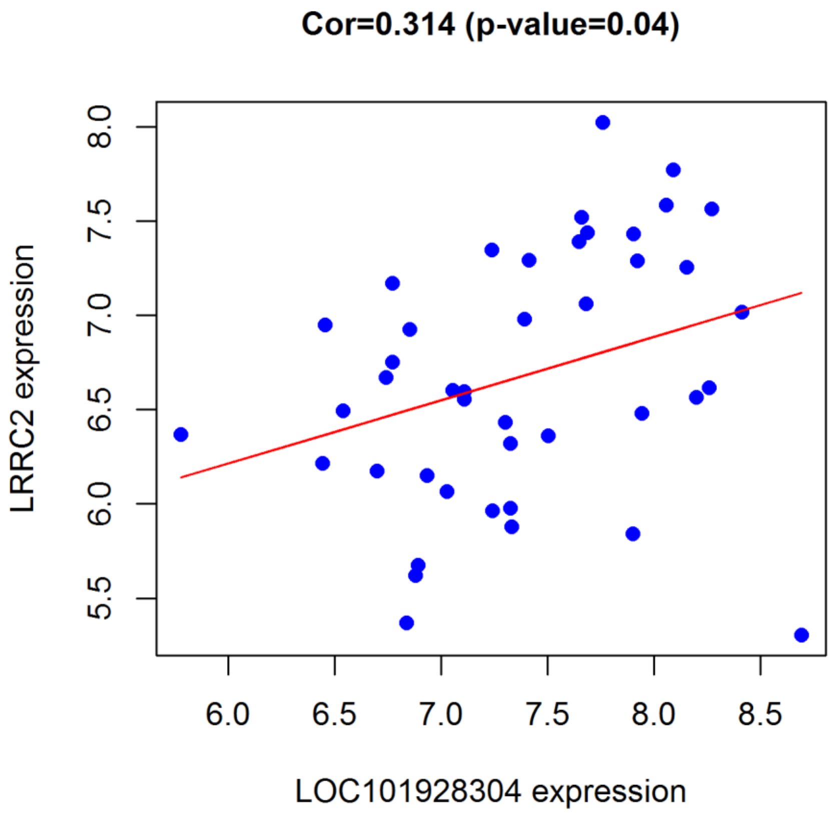

Supplementary Figure 2 LOC101928304 was positively correlated with LRRC2.
